# The Epidemiology of Herpes Simplex Virus Type-2 Infection among Pregnant Women in Rural Mysore *Taluk,* India

**DOI:** 10.1155/2013/750415

**Published:** 2013-11-14

**Authors:** Aaron F. Bochner, Purnima Madhivanan, Bhavana Niranjankumar, Kavitha Ravi, Anjali Arun, Karl Krupp, Jeffrey D. Klausner

**Affiliations:** ^1^Department of Epidemiology, School of Public Health, University of Washington, Seattle, WA 98195, USA; ^2^Department of Epidemiology, Robert Stempel College of Public Health and Social Work, Florida International University, 11200 SW 8 Street, HLS 390W2, Miami, FL 33199, USA; ^3^Public Health Research Institute of India, Mysore, Karnataka 560020, India; ^4^Department of Health Promotion and Disease Prevention, Robert Stempel College of Public Health and Social Work, Florida International University, Miami, FL 33199, USA; ^5^Division of Infectious Diseases, David Geffen School of Medicine, University of California, Los Angeles, CA 90095, USA

## Abstract

*Objectives*. To assess the prevalence and determinants of herpes simplex virus type 2 (HSV-2) infections among pregnant women attending mobile antenatal health clinic in rural villages in Mysore *Taluk*, India. *Methods*. Between January and September 2009, 487 women from 52 villages participated in this study. Each participant consented to provide a blood sample for HSV-2 and HIV testing and underwent an interviewer-administered questionnaire. *Results*. HSV-2 prevalence was 6.7% (95% confidence interval (CI) 4.4–9.0), and one woman tested positive for HIV. The median age of women was 20 years and 99% of women reported having a single lifetime sex partner. Women whose sex partner traveled away from home had 2.68 (CI: 1.13–6.34) times the odds of being HSV-2 seropositive compared to women whose sex partner did not travel. Having experienced genital lesions was also associated with HSV-2 infection (*P* value = 0.08). *Conclusion*. The 6.7% HSV-2 prevalence was similar to results obtained in studies among pregnant women in other parts of India. It appeared that most women in this study contracted HSV-2 from their spouses and few regularly used condoms. This finding highlights the need for public health policies to increase awareness and education about prevention methods among women and men living in rural India.

## 1. Background

An estimated 536 million people aged 15–49 are infected with the herpes simplex virus type 2 (HSV-2) worldwide [1]. HSV-2 is typically spread through sexual contact and results in a lifelong infection. The predominant symptom of the disease is genital lesions, but a majority of infected individuals experience no symptoms or mild ones that are often unrecognized [2]. The high rate of asymptomatic cases enhances HSV-2 transmission because asymptomatic individuals shed the virus and transmit the disease [3].

Pregnant women are particularly vulnerable to the adverse sequelae of HSV-2 infection [4]. HSV-2 infection in pregnancy has been associated with premature delivery, low birthweight infants, fetal malformations, and vertical transmission of the virus during childbirth [4, 5]. While neonatal herpes occurs in less than 1% of prevalent infections, the risk of transmission increases to 25–50% among women infected during pregnancy [6, 7]. In addition, it is estimated that prevalent HSV-2 infection is associated with a 2- to 4-fold increased risk of HIV-1 acquisition [8].

The seroprevalence of HSV-2 varies by region, country, and population [9]. Various studies have estimated that about 40% of pregnant women in sub-Saharan Africa, 30% in Latin America, 17–22% in North America, and 4 to 24% in Europe are HSV-2 infected [8, 10–12]. An analysis of prevalence by country shows even greater heterogeneity. Studies from Sweden, Italy, Switzerland, and China show the HSV-2 prevalence in antenatal cases of 10.4%, 7.6%, 20.7%, and 10.8%, respectively [10, 13–15]. 

There are few studies describing HSV-2 prevalence among pregnant women in India. One cross-sectional study conducted among 1,640 pregnant women attending antenatal clinics in Arunachal Pradesh, Assam, Manipur, Meghalaya, and Mizoram (all states are located in the northeast of India) found overall HSV-2 seroprevalence of 8.7% with states ranging from a high of percentage 15% in Arunachal Pradesh to a low percentage of 2.7% in Manipur [16]. Another study of asymptomatic pregnant females attending the Obstetrics and Gynecology Outpatient Department for a routine antenatal checkup in the North Indian State of Jammu found HSV-2 prevalence of 7.5% [17].

With one antenatal study from Tamil Nadu, a southern state in India, and limited seroprevalence estimates from other parts of the country, there is a great need for additional studies among pregnant women [18]. The research describes the prevalence and determinants of HSV-2 infection among pregnant women living in rural villages in Mysore *Taluk*, Karnataka, India.

## 2. Methods

### 2.1. Setting

Public Health Research Institute of India (PHRII) is a nonprofit organization based in Mysore, India. PHRII operates a health clinic in Mysore providing free reproductive healthcare to women. PHRII maintains a public health laboratory and conducts mobile health clinics offering integrated antenatal care and HIV testing services in villages in Mysore *Taluk* for pregnant women. Mysore *Taluk* is located in the southern part of the state of Karnataka, India.

### 2.2. Study Population

Pregnant women living in rural villages located in Mysore *Taluk* were enrolled in the study from January to September 2009. Pregnant women in the villages were recruited through extensive community outreach and with the assistance of community and local health professionals. A total of 487 women from 52 villages submitted blood samples to be tested for HSV-2 and HIV. The Institutional Review Boards at the University of California, Berkeley, and Public Health Research Institute of India approved the study protocol.

### 2.3. Study Design

A list of the 141 villages in Mysore *Taluk* was obtained through the 2001 Indian Census and 52 villages were randomly selected to participate in an intervention providing integrated antenatal care and HIV testing using mobile medical clinics. Prior to the health camp, an outreach team met with village leaders and local health professionals to seek permission to work in the community. The team also met with the national rural health mission workers—the auxiliary nurse midwife (ANM), the accredited social health activist (ASHA), *Anganwadi* teacher and helper to discuss the program. The *Anganwadi* teacher provided a list of registered pregnant women in each of the villages and assisted in referring these women to the health clinic. Mobile health clinics were typically conducted in primary schools or community halls located in each village one day after a community-wide education and awareness program was conducted on the importance of antenatal care and institutional deliveries. Pregnant women were offered a free antenatal screening including clinical examination and laboratory evaluation and completed an interviewer-administered questionnaire. 

### 2.4. Interview and Counseling

All pregnant women who came to the mobile medical clinic were offered pretest counseling in groups in the local language of *Kannada*. Each woman was then directed to a private space where she could discuss and ask any questions or clarifications before proceeding further through the program. Then, a trained female interviewer used a standardized questionnaire to collect information on sociodemographic status, work experience and economic status, knowledge about HIV/AIDS, sexual history, information on main sex partners, history of sexually transmitted infections, and HIV test reports. All women were assured of confidentiality of their responses. 

### 2.5. Diagnostic Testing

After completion of an examination by the physician, a trained nurse collected blood samples from consenting participants to screen for HSV-2, HIV, and other routine antenatal investigations. Diagnostic tests were performed in the PHRII laboratory in Mysore. A type-specific ELISA was used to detect HSV-2 IgG antibodies in sera (Focus Technologies, Cypress, CA, USA). Tests were performed following manufacturer instructions using index values > 1.1 to define positive results. A blinded panel of 20 serum samples provided by St. John's Medical College Research Institute, Bangalore, was used to validate testing and results were in agreement with 20 out of 20 samples. The first 89 samples were run in duplicate and all results were successfully replicated. In addition, all positive test results were duplicated to provide confirmation. HIV testing was performed using ELISA following manufacturer instructions (HIVASE 1 + 2, General Biologicals Corporation, Taiwan). All HIV testing was performed in duplicate. Any woman who was found to be infected with HIV was provided medications for prevention of vertical transmission of HIV and referred to the antiretroviral therapy center in KR Hospital. All women were subsequently followed up after delivery up to a period of two years.

### 2.6. Statistical Analysis

Data were analyzed using Stata 10.1 (Stata Corporation, College Station, TX). Pearson's chi-square test adjusted to account for the cluster survey design was used to assess association of clinical characteristics and HSV-2 status using a *P* value cutoff of 0.1 for significance [19]. To obtain odds ratios, data were modeled using logistic regression, with HSV-2 status as the binary outcome of interest. In addition, relative risks were obtained using a generalized linear model with a log link function and binomial distribution. A cluster survey design was used because pregnant women were geographically grouped into villages; thus robust variance estimates adjusted within village correlation were obtained [19]. 

## 3. Results

Most of the pregnant women in the selected villages participated in the program. Only 21 pregnant women in the community did not provide a blood sample, either because they had only recently done their antenatal investigations (*n* = 8) or they declined to attend the medical clinic for personal reasons (*n* = 13). Of the 487 (95.6%) pregnant women tested for HSV-2 antibodies, 32 tested positive, giving a prevalence of 6.6%. Accounting for the cluster design of the sampling gives a prevalence of 6.7% (95% confidence interval (CI): 4.4–9.0). Of these 487 women, only one woman (0.2%) tested positive for human immunodeficiency virus antibodies.

Of the 487 women who provided a blood sample, 478 (98%) agreed to be interviewed, which included all women who tested positive for HSV-2. Characteristics of the study population are shown in Table 1. The median age of the study population was 20 years, ranging from 15 to 33 years of age. Education level varied, with 13% of women reporting completion of less than one year of education, while 33% had completed 1–7 years, 36% had completed 8–10 years, and 18% had completed more than 10 years of school. All but one woman participating in the study were married, with 34% of women married for one year or less, 40% married between 1 and 3 years, and only 26% married for longer than three years. About half the women in the study had children (52%). The median household income per month was 3000 INR (1 USD = 45.9 INR), ranging from 0 to 40,000 INR per month, while only 2.7% (13/478) of participants reported regularly earning income themselves. Almost all of the participants (99.2%, 473/477) in the study population reported a single sex partner in their lifetime and 88.7% (422/476) of study participants reported never using a condom with their main sex partner. 

The majority of participants who tested positive for HSV-2 did not report symptoms commonly associated with HSV-2 (Table 2). Vaginal sores were experienced by 4% of the women in the study, while 21% reported experiencing painful urination and 13% reported having abnormal vaginal discharge. Evidence of an association was found for the presence of vaginal sores (*P* value = 0.08), with 9% of HSV-2 seropositive women reporting vaginal sores compared to 3% of HSV-2 seronegative women. Painful urination (*P* value = 0.26) and abnormal vaginal discharge (*P* value = 0.49) were not associated with HSV-2 status.

Using logistic regression, the unadjusted odds ratio was determined for each characteristic (Table 3). The only characteristic that showed evidence of affecting the odds of HSV-2 infection was if the sex partner ever traveled away from home, where participants whose partner ever traveled had 2.68 times the odds of being HSV-2 positive as compared to participants whose partner never traveled (95% CI: 1.14–6.34). A generalized linear model estimated the relative risk of HSV-2 among women whose partners ever traveled to be 2.44 (95% CI: 1.16–5.14) times greater than the probability of women whose husbands never traveled. 

To control for possible confounding, multivariable logistic regression was used to adjust for the participant's age and husband's education level. In this model, women whose main sex partner ever traveled away from home had 2.77 (95% CI: 1.20–6.41) times the odds of having a prevalent HSV-2 infection. 

## 4. Discussion

This study found HSV-2 prevalence of 6.7% among pregnant women attending mobile antenatal care clinics in 52 rural villages located in Mysore *Taluk*, Karnataka, India. This is comparable to previously obtained measures of HSV-2 prevalence among other pregnant women in India [16, 17]. The 6.7% prevalence is lower than the three previously obtained estimates of HSV-2 prevalence in the state of Karnataka, which ranged from 10.9 to 18.9% [20–22] although these studies focused on nonpregnant populations.

Almost all the women in this study reported having a single sex partner in their lifetime and one woman reported being unmarried. It appears that the main route of HSV-2 transmission in this population is through sexual transmission from their spouses. Since very few married women consistently use a condom, the risk behavior of their husbands is a primary risk factor. This information highlights the need to increase awareness and discuss risk behaviors among both men and women living in rural India.

In our exploratory analysis, only having a husband who traveled away from home was significantly associated with an increased risk of HSV-2 infection. *P* values were not adjusted for multiple comparisons. Misclassification should not have affected this finding since women would know how often their husbands traveled and there is no social stigma attached to this activity. In addition, living in a small rural village may present limited opportunity for sex outside of marriage, so only husbands who travel may have access to other sex partners. Furthermore, this may be the main entry point for HSV-2 into rural communities since sex workers are more common in urban areas and have been shown to have high rates of HSV-2 infection [23]. 

It is possible that this study overestimated the prevalence of HSV-2 in this population. There is a debate about the accuracy of the Focus Diagnostics HerpeSelect HSV-2 IgG ELISA test kits used in this study. Some researchers have suggested that the cutoff value for a positive test should be raised from 1.1 to 3.0 or 3.5 when the test is used in low prevalence settings [24, 25]. Raising the cutoff value to 3.5 would have affected the study results since 14 of the 32 positive tests fell in the disputed range, decreasing the estimated prevalence from 6.6% to 4.7%. Additional HSV-2 studies should be conducted using a gold standard like western blot to confirm the results.

In this study, the majority of HSV-2 positive individuals did not report experiencing symptoms caused by HSV-2. This is consistent with previous findings that HSV-2 is frequently asymptomatic or symptoms go unrecognized. We did find an association between the presence of vaginal sores and HSV-2 (*P* value = 0.08). However, only 9.4% of HSV-2 positive women reported experiencing vaginal sores, highlighting the challenge of diagnosing HSV-2 and the ability of HSV-2 to circulate undetected in a population. 

There are several limitations to the study. Information about spouses was obtained from participants who were likely to possess incomplete knowledge of their partners' behaviors, resulting in misclassification. There is the possibility of social desirability bias. Though interviews were conducted in a private setting and women were guaranteed anonymity, it is possible that participants still feared disclosing personally or socially sensitive information. This may also have resulted in misclassification that biased the results toward finding no association between certain risk behaviors. This study was limited by a small sample size and a low prevalence of HSV-2 in the study population limiting the power of the study to detect associations between personal characteristics and HSV-2. Associations found in other studies between older age and alcohol consumption and increased risk for HSV-2 infection were not found to be statistically significant in this study. It is possible that with a larger sample size, associations would be found between additional factors and increased risk of HSV-2 infection. 

This study provides needed information regarding the prevalence of HSV-2 among rural pregnant women in India and will serve as an important baseline measurement as investigators continue to monitor rates of HSV-2 in the future in rural India.

## Figures and Tables

**Table 1:** Descriptive characteristics of the sample of pregnant women attending the mobile medical clinics in rural Mysore, India.

Characteristic	HSV-2			
	Seronegative		Seropositive	
	No.	(%)	No.	(%)
Age	446		32	
≤17	15	28.6%	0	0%
18–20	228	60.0%	15	46.8%
21–23	142	32.1%	11	34.4%
≥24	61	13.9%	6	18.8%
Education completed, years	445		32	
None	60	13.5%	4	12.5%
1–7	144	32.4%	14	43.8%
8–10	165	37.1%	6	18.8%
>10	76	17.1%	8	15.0%
Length of marriage, years	446		32	
≤1	151	33.9%	10	31.3%
1–3	181	40.6%	11	34.4%
>3	114	25.6%	11	34.4%
Have children	446		32	
No	248	55.6%	19	59.4%
Yes	198	44.4%	13	40.6%
Household income, INR/month	445		32	
<4000 ($87 USD)	232	52.1%	18	56.3%
≥4000 ($87 USD)	213	47.9%	14	43.8%
Sex while you or partner was under influence of alcohol	441		32	
Never	386	87.5%	26	81.3%
Ever	55	12.5%	6	18.8%
Main partner smokes cigarettes	439		32	
Never	288	65.6%	23	71.9%
Ever	151	34.4%	9	28.1%
Main partner education completed, years	443		32	
None	119	26.9%	10	31.3%
1–7	115	26.0%	9	28.1%
8–10	130	29.4%	6	18.8%
>10	79	17.8%	7	21.9%
Main partner travels	444		32	
Never	402	90.5%	25	78.1%
Ever	42	9.5%	7	21.9%
Main partner had other sex partners	444		32	
Never	374	84.2%	25	78.1%
Ever	48	10.8%	3	9.4%
Do not know/refused	22	5.0%	4	12.5%

**Table 2:** Clinical characteristics of the sample of pregnant women living in rural Mysore *Taluk*, India.

Characteristics	HSV-2				Design-based Pearson *P* value
	Seronegative (*n* = 443)		Seropositive (*n* = 32)		
	No.	(%)	No.	(%)	
Vaginal sores					0.077
Ever	14	3.1%	3	9.4%	
Never	429	96.8%	29	90.6%	
Painful urination					0.258
Ever	89	20.1%	9	28.1%	
Never	354	79.9%	23	71.9%	
Abnormal vaginal discharge∗					0.492
Ever	55	12.4%	5	15.6%	
Never	387	87.6%	27	84.4%	

^*^One individual with missing data.

**Table 3:** Correlates of HSV-2 infection among pregnant women living in rural Mysore *Taluk*, India.

Characteristic	HSV-2 seropositivity			
	OR	95% CI	RR	95% CI
Age				
15–19	1.00		1.00	
20–24	1.19	(0.49, 2.89)	1.18	(0.51, 2.71)
>25	1.51	(0.49, 4.67)	1.47	(0.52, 4.16)
Education completed, years				
None	1.00		1.00	
1–7	1.46	(0.45, 4.76)	1.42	(0.47, 4.27)
8–10	0.55	(0.17, 1.81)	0.56	(0.18, 1.75)
>10	1.58	(0.42, 5.95)	1.52	(0.45, 5.20)
Length of marriage, years				
<1	1.00		1.00	
1–3	0.92	(0.42, 2.02)	0.92	(0.44, 1.93)
>3	1.46	(0.70, 3.04)	1.42	(0.72, 2.79)
Have children				
No	1.00		1.00	
Yes	0.86	(0.38, 1.96)	0.87	(0.40, 1.87)
Household income, INR/month				
<4000	1.00		1.00	
>4000	0.85	(0.35, 2.04)	0.86	(0.38, 1.95)
Sex while you or partner was under influence of alcohol				
Never	1.00		1.00	
Ever	1.62	(0.67, 3.92)	1.56	(0.70, 3.48)
Main partner smokes cigarettes				
Never	1.00		1.00	
Ever	0.75	(0.34, 1.64)	0.76	(0.36, 1.59)
Main partner education completed, years				
None	1.00		1.00	
1–7	0.93	(0.36, 2.44)	0.94	(0.38, 2.28)
8–10	0.55	(0.19, 1.58)	0.57	(0.21, 1.54)
>10	1.05	(0.43, 2.60)	1.05	(0.46, 2.41)
Main partner travels				
Never	1.00		1.00	
Ever	**2.68**	**(1.13, 6.34)** ∗	**2.44**	**(1.16, 5.14)** ^~^
Main partner had other sex partners				
No	1.00		1.00	
Yes	0.94	(0.28, 3.15)	0.94	(0.30, 2.95)
Do not know/refused	2.72	(0.89, 8.27)	2.46	(0.94, 6.43)

^*^ *P* value = 0.026.

^~^ *P* value = 0.020.
